# Disease Control and Toxicity Outcomes after Stereotactic Ablative Radiation Therapy for Recurrent and/or Metastatic Cancers in Young-Adult and Pediatric Patients

**DOI:** 10.3390/cancers16112090

**Published:** 2024-05-30

**Authors:** Rituraj Upadhyay, Brett Klamer, Jennifer Matsui, Vikram B. Chakravarthy, Thomas Scharschmidt, Nicholas Yeager, Bhuvana A. Setty, Timothy P. Cripe, Ryan D. Roberts, Jennifer H. Aldrink, Raj Singh, Raju R. Raval, Joshua D. Palmer, Sujith Baliga

**Affiliations:** 1 Department of Radiation Oncology, The Ohio State University Wexner Medical Center, Columbus, OH 43210, USA; rituraj.upadhyay@osumc.edu (R.U.); jennifer.matsui@osumc.edu (J.M.); raj.singh@osumc.edu (R.S.); raju.raval@osumc.edu (R.R.R.); joshua.palmer@osumc.edu (J.D.P.); 2 Center for Biostatistics, The Ohio State University Wexner Medical Center, Columbus, OH 43210, USA; brett.klamer@osumc.edu; 3 Department of Neurosurgery, The Ohio State University Wexner Medical Center, Columbus, OH 43210, USA; vikram.chakravarthy@osumc.edu; 4 Department of Orthopedic Surgery, Nationwide Children’s Hospital, Columbus, OH 43215, USA; thomas.scharschmidt@osumc.edu; 5 Department of Pediatric Oncology, Nationwide Children’s Hospital, Columbus, OH 43205, USA; nicholas.yeager@nationwidechildrens.org (N.Y.); bhuvana.setty@nationwidechildrens.org (B.A.S.); timothy.cripe@osumc.edu (T.P.C.); roberts.668@osu.edu (R.D.R.); 6 Division of Pediatric Surgery, Department of Surgery, Nationwide Children’s Hospital, The Ohio State University College of Medicine, Columbus, OH 43205, USA; jennifer.aldrink@nationwidechildrens.org

**Keywords:** pediatric cancer, SABR, stereotactic body radiotherapy, systemic therapy, metastatic cancer

## Abstract

**Simple Summary:**

Pediatric patients with recurrent and metastatic cancers often present with substantial tumor and symptom burden. Local control is an important factor to consider in this setting. Stereotactic ablative radiation therapy (SABR) offers a therapeutic advantage with higher, ablative doses potentially providing durable local control and a shorter fractionation schedule allowing minimum interruptions in systemic therapy and disruption in quality of life. In this study, we evaluate the outcomes of pediatric patients treated with SABR. We observed that SABR is well tolerated with local failure rates of <10% at 1 year and a median survival of 16.9 months. Patients with oligometastatic disease had a better survival rate than patients with widely metastatic disease, suggesting that the total consolidation of all metastatic sites in patients with a limited metastatic burden may be associated with better survival outcomes. Higher local control was associated with a higher radiation dose and sarcoma histology. Future studies evaluating SABR in combination with systemic therapy are warranted.

**Abstract:**

Background: Pediatric patients with metastatic and/or recurrent solid tumors have poor survival outcomes despite standard-of-care systemic therapy. Stereotactic ablative radiation therapy (SABR) may improve tumor control. We report the outcomes with the use of SABR in our pediatric solid tumor population. Methods: This was a single-institutional study in patients < 30 years treated with SABR. The primary endpoint was local control (LC), while the secondary endpoints were progression-free survival (PFS), overall survival (OS), and toxicity. The survival analysis was performed using Kaplan–Meier estimates in R v4.2.3. Results: In total, 48 patients receiving 135 SABR courses were included. The median age was 15.6 years (interquartile range, IQR 14–23 y) and the median follow-up was 18.1 months (IQR: 7.7–29.1). The median SABR dose was 30 Gy (IQR 25–35 Gy). The most common primary histologies were Ewing sarcoma (25%), rhabdomyosarcoma (17%), osteosarcoma (13%), and central nervous system (CNS) gliomas (13%). Furthermore, 57% of patients had oligometastatic disease (≤5 lesions) at the time of SABR. The one-year LC, PFS, and OS rates were 94%, 22%, and 70%, respectively. No grade 4 or higher toxicities were observed, while the rates of any grade 1, 2, and 3 toxicities were 11.8%, 3.7%, and 4.4%, respectively. Patients with oligometastatic disease, lung, or brain metastases and those who underwent surgery for a metastatic site had a significantly longer PFS. LC at 1-year was significantly higher for patients with a sarcoma histology (95.7% vs. 86.5%, *p* = 0.01) and for those who received a biological equivalent dose (BED10) > 48 Gy (100% vs. 91.2%, *p* = 0.001). Conclusions: SABR is well tolerated in pediatric patients with 1-year local failure and OS rates of <10% and 70%, respectively. Future studies evaluating SABR in combination with systemic therapy are needed to address progression outside of the irradiated field.

## 1. Introduction

Pediatric cancers often present in the advanced and/or metastatic stage. With the development of novel systemic therapy regimens combined with technological advances in radiation techniques and surgery, children and adolescent and young-adult (AYA) patients with cancer have excellent outcomes with a 5-year overall survival (OS) of over 85% [1]. However, the prognosis remains poor for patients with advanced and/or metastatic disease [2,3]. Local or distant recurrence is a major cause of morbidity and mortality in these patients, accounting for two-thirds of all deaths in 5-year survivors [4]. Palliative radiotherapy is often effective for the alleviation of symptoms but often does not provide durable local control (LC) [5,6]. In particular, certain solid tumors such as bone and soft tissue sarcomas and central nervous system tumors are considered radioresistant [7,8,9]. Stereotactic ablative radiation therapy (SABR) is a non-invasive radiation technique that allows the delivery of higher, ablative radiation doses in 1–5 fractions [10,11]. The resulting higher biological equivalent dose (BED) may improve tumor control [12], especially in recurrent/metastatic settings in which the tumors are often refractory to other therapies or for tumors in eloquent areas that present a challenge for the delivery of conventionally fractionated radiotherapy [13].

Recent data have suggested an improvement in LC as well as OS with the use of SABR in the adult population [14,15], but the impact of SABR in pediatric patients has not been very well elucidated in the literature. There are several advantages of SABR for pediatric patients, including fewer visits requiring anesthesia, fewer interruptions in resuming systemic therapy, and minimum time away from family and community. Our previous study with 16 patients demonstrated the safety and efficacy of SBRT in pediatric and adolescent patients, albeit with a limited sample size [16]. To better define the role of SABR in pediatric patients, we reviewed the LC, OS, and toxicity outcomes with the use of SABR in our pediatric and adolescent and young-adult (AYA) population in a larger cohort of patients with a longer follow-up, with the primary aim of identifying the impact of the radiation dose on LC.

## 2. Materials and Methods

### 2.1. Patient Selection and Study Design

We included pediatric and AYA patients < 30 years of age treated with SABR at our National Cancer Institute-designated Cancer Center from January 2016 to December 2022. This study was approved by our institutional review board (IRB approval number 2023C0242). Patients who were treated with radiation doses of ≥5 Gy per fraction in 5 or less fractions were included. Patients were excluded if they had <3 months of follow-up data or if they received fractionated radiation therapy in >5 fractions.

All the patients underwent a comprehensive pre-treatment evaluation, which included a detailed medical history, physical examination, lab studies including complete blood cell count (CBC) and complete metabolic profile (CMP), and appropriate radiographic studies to assess disease extent. All the patients’ cases were discussed in a multidisciplinary conference. Oligometastatic disease was defined as the presence of ≤5 lesions at the time of SABR.

### 2.2. Radiation Treatment Planning

**Figure 1:**
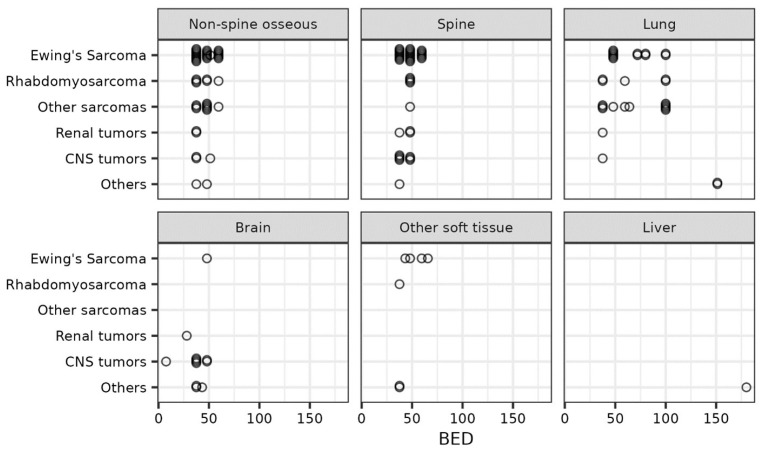
Scatter plot depicting the biological equivalent dose (BED) of radiation delivered by treatment site and primary tumor histology.

The patient setup was dependent on the treatment site and has been described in detail in our prior publication [16]. The gross tumor volume (GTV) was defined as the gross disease seen on a clinical exam or radiographic imaging. An internal target volume (ITV) was generated for metastatic sites in the lung, liver, or abdomen using 4DCTs to account for tumor motion. The clinical target volume (CTV) margin varied with the site, with no CTV used for most sites, but a margin of 3–5 mm was allowed based on clinical judgement. A planning target volume (PTV) margin of 0–5 mm was used to account for setup errors. No PTV margin was used for spinal metastases [17]. Most patients were treated with volumetric arc therapy (VMAT) with full arcs for centrally located targets while partial arcs were chosen for unilateral target locations. All patients were treated with photons. The prescribed dose was dependent on the tumor location and histology. The BED was calculated assuming an α/β ratio of 10 for the tumor (BED_10_). Figure 1 depicts the BED_10_ scatter plot describing prescriptions doses for various tumor locations and histologies. Treatment was delivered daily with a cone-beam CT supervised by the treating radiation oncologist.

Treatment planning was carried out by inverse optimization using VMAT to allow 95% of the PTV to receive the prescription dose, with a steep dose gradient to achieve sparing of nearby organs at risk. A hot spot of 110–150% of the prescription dose was acceptable depending on the disease site and surrounding normal tissue. A simultaneously integrated boost (SIB) approach was used to achieve a 12–25% hot spot in the GTV as feasible. A single-isocenter multitarget (SIMT) technique was used if multiple targets could be treated with a single isocenter using the appropriate collimator and gantry angles. The dose constraints were based on the most recent available Children’s Oncology Group Trials, for example ARST 1431 for rhabdomyosarcoma, and American Association of Physicists in Medicine (AAPM) Task Group 101 guidelines by treatment site. The typical normal tissue constraints for central nervous system normal structures were a maximum point dose of 27 Gy for the spinal cord, 30 Gy for the thecal sac, and 28.8 Gy for the sacral plexus and cauda equina. For lung SABR planning, we aimed to keep 1500 cc of the right and left lung below 11.2 Gy and the trachea and ipsilateral bronchus to 34.2 Gy maximum point dose. For SABR to lower-extremity osseous metastases, we constrained the femoral heads to a volume of <10 cc receiving 27 Gy.

### 2.3. Follow-Up and Response Assessment

The patients were followed with clinical examination and imaging (contrast-enhanced computed tomography (CT), magnetic resonance imaging (MRI), and/or PET/CT) every 2–3 months after completing SABR. Patients alive at the time of analysis were censored on the date of their last clinic visit or imaging. Local progression was defined as progression of the tumor within the radiation-treated field, while any failure outside the radiation field was considered distant progression. LC was defined as the time between end of SABR to date of local progression where death without local progression was a competing event and subjects alive without local progression at last known follow-up were censored. Progression-free survival (PFS) was defined as time to local or distant progression or death with censoring of subjects without progression at last known follow-up. OS was defined as time to death from any cause with censoring of patients alive at last follow-up. Patients who were lost to follow-up were censored at that timepoint. OS analysis was performed on a per-patient basis while PFS and LC were evaluated on a per-course basis (assuming multiple observations per patient were uncorrelated). Treatment response was assessed using the modified Response Evaluation Criteria in Solid Tumors criteria (RECIST version 1.1) [18]. Adverse events were recorded and graded according to the Common Terminology Criteria for Adverse Events version 5.0 (CTCAE v5.0) acute and late toxicity grading scales [19].

### 2.4. Statistical Analysis

The patient characteristics are presented as median and interquartile ranges (IQRs) for continuous data and as frequency (percent) for categorical data. The Pearson chi-squared test was used for categorical variables and the Wilcoxon rank-sum test for continuous variables to assess measures of association in frequency tables. For local failure, cumulative incidences were calculated using death without local failure as a competing event, and intergroup comparison was carried out using Fine and Gray competing-risks univariable regression model. The endpoints with respect to OS and PFS were calculated by the Kaplan–Meier method, and the log-rank test was used for intergroup comparison. Univariate (UVA) and multivariable (MVA) analyses using Cox proportional hazards models were conducted to evaluate the associations between pertinent clinical factors and outcomes. Age, sex, and other independent variables with a *p*-value < 0.05 on UVA were included in the MVA predictive model. Both Cox proportional hazards and Fine and Gray competing-risks regression analyses are presented using hazard ratios (HRs) and their 95% confidence intervals (CIs). *P*-values < 0.05 were considered statistically significant. The statistical tests were based on a 2-sided significance level. All the statistical analyses were performed using SPSS v23.0 (IBM Corp, Armonk, NY, USA) and R version 4.2.3. (R Core Team, R Foundation for Statistical Computing) with the survival and cmprsk packages.

## 3. Results

A total of 48 patients who received 135 courses of SABR were included in this study. Table 1 describes the baseline patient and tumor characteristics of our cohort. The median follow-up time was 18.1 months (IQR: 7.7–29.1). The median age at the time of SABR was 15.6 years (IQR: 14–23). The most common primary histologies were Ewing sarcoma (25%), rhabdomyosarcoma (17%), osteosarcoma (13%), and CNS gliomas (13%). The most common sites for SABR were extraspinal osseous metastases (33%) followed by the spine (29%), lung (24%), and CNS (9%). Most patients received the treatment in five fractions, with a median total dose of 30 Gy (IQR: 25–35). The median BED_10_ was 48 Gy (IQR: 38–60), with 37 (27%) courses receiving > 48 Gy and 12 (9%) courses receiving ≥100 Gy. Patients with lung metastases received a higher median BED_10_ of 62 Gy (IQR: 48–100) compared to 48 Gy (IQR: 38–48) for those with bone tumors and 38 Gy (IQR: 38–45) for brain tumors. The three histologies associated with the highest BED_10_ were adenoid cystic carcinoma, synovial sarcoma, and osteosarcoma (Figure 1).

### 3.1. Local Control

**Figure 2:**
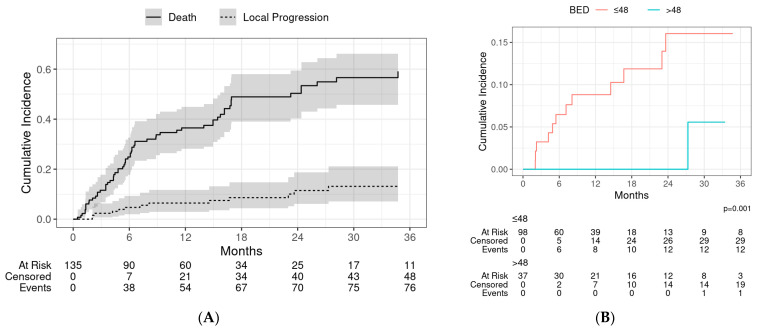
(**A**) Fine and Gray competing risk model of local progression with death without local progression as a competing event. Shaded regions represent 95% confidence intervals at each timepoint. (**B**) Local progression stratified by biological equivalent dose (BED).

Thirteen patients had a local failure, with a crude incidence of 9.6%. The cumulative incidence of local progression at 1 and 2 years was 6.4% (95% CI: 3.0–12.0) and 11.0% (95% CI: 6.1–19.0), respectively (Figure 2A). On stratifying by the median BED_10_, local control at 1 year and 2 years, respectively, was 100% and 100% for patients who received a BED_10_ > 48 Gy, and 91.2% and 84% for patients who received BED_10_ < 48 Gy (*p* = 0.001) (Figure 2B). No local failures were observed in patients who received a BED_10_ ≥ 100 Gy.

Table 2 describes the UVA and MVA of variables affecting local control. On UVA, local control was significantly improved for patients with higher age at SABR (HR of local progression = 0.88, *p* = 0.008), sarcoma histology (HR of local progression = 0.24, *p* = 0.010), and higher BED_10_ (HR of local progression = 0.37, *p* = 0.001). Higher BED_10_ remained a significant predictor of LC on MVA after controlling for age, sex, and prior radiation (HR of local progression = 0.44, *p* < 0.001). None of the patients who received a BED_10_ of >100 Gy had a local failure. LC was also poorer in patients who received concurrent chemotherapy during SABR. LC at 1 year was significantly higher for patients with a sarcoma histology (95.7% vs. 86.5%, *p* = 0.01; Supplementary Figure S1). There was no difference in local control by treated tumor site (Supplementary Figure S2).

### 3.2. Overall Survival and Progression-Free Survival

**Figure 3:**
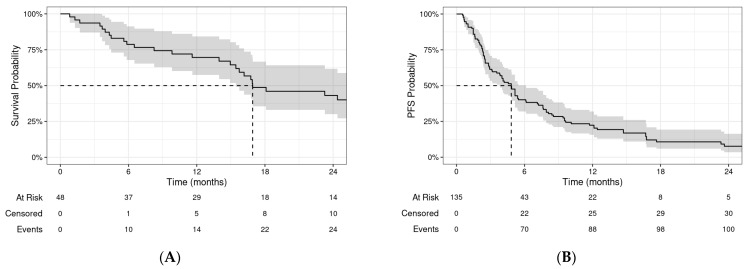
Kaplan–Meier curves depicting (**A**) overall survival (OS) and (**B**) progression-free survival (PFS) for all patients. Shaded regions represent 95% confidence intervals at each timepoint.

The median OS was 16.9 months (95% CI: 15.4–34.7) for the entire cohort (Figure 3A). The 1-year and 2-year OSs were 70% (95% CI: 57–84%) and 43% (95% CI: 30–62%), respectively. On UVA, OS improved with increasing age, female sex, higher Lansky performance score (PS), oligometastatic disease, BED_10_ > 48 Gy, previous RT, and no concurrent chemotherapy. Only age, PS, and oligometastatic disease remained significant predictors on MVA (Supplementary Table S1).

**Figure 4:**
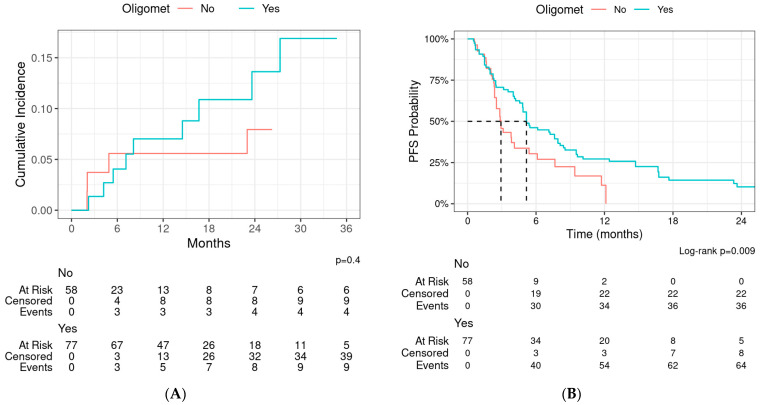
(**A**) Local progression and (**B**) progression-free survival (PFS)—stratified by oligometastatic disease.

The median PFS was 4.8 months (95% CI: 3.8–6.1) by course (Figure 3B). The 1-year and 2-year PFSs were 22% (95% CI: 16–32%) and 8% (95% CI: 4–16%), respectively. Patients with oligometastatic disease had an improved progression-free survival compared to those without (*p* = 0.009, Figure 4). On UVA, there was significant association of PFS with treatment site (lung and brain with improved PFS), previous radiation (HR for progression 0.57, *p* = 0.025), oligometastatic disease (HR for progression 0.57, *p* = 0.011), and surgery for metastases (HR for progression 0.58, *p* = 0.040). None of the variables were associated with PFS on MVA after controlling for age and sex (Supplementary Table S2).

### 3.3. Adverse Events

The rates of any grade 1, 2, and 3 toxicities were 11.8%, 3.7%, and 4.4%, respectively. The most common acute toxicity was grade 1 dermatitis, while the most common late toxicity was grade 1 chronic pneumonitis. No patients had acute grade 3+ toxicity. Six patients had late grade 3 toxicity, including two with chronic pneumonitis (BED_10_ 100 Gy to lung metastases), two with rib myositis (BED_10_ 64 and 48 Gy to lung metastases), and one each with neuropathy (BED_10_ 37.5 Gy to pelvis) and brachial plexopathy (BED_10_ 59.5 Gy to the head of humerus). Only one of these six patients received concurrent systemic therapy (vs. 30% of the entire cohort). No grade 4 or higher toxicities were observed. The toxicities per course are described in detail in Supplementary Table S3.

## 4. Discussion

Pediatric patients with recurrent and metastatic cancers can have substantial local tumor burden and resulting symptoms. In this setting, local tumor control is important in alleviating symptoms and preventing progression and can have a significant impact on the quality of life of these patients. SABR offers a therapeutic advantage with higher, ablative doses potentially providing durable local control even for relatively radioresistant histologies and a shorter fractionation schedule allowing minimum interruptions in systemic therapy and disruption in quality of life. We report here one of the largest series evaluating the outcomes of pediatric and AYA patients treated with SABR. We have previously presented our pilot data describing outcomes with 16 patients receiving SABR [16]. We observed that SABR is well tolerated in these patients with local failure rates of 6.4% at 1 year and a median OS of 16.9 months.

We observed 1- and 2-year LC rates of 93.6% and 89.0%, respectively, in our cohort. A recent meta-analysis of nine studies (LITE-SABR meta-analysis) evaluating a total of 142 pediatric patients reported estimated 1- and 2-year LC rates of 83.5% and 74.0%, respectively [20]. Only one prospective trial to date has reported local control outcomes after SABR for pediatric patients [21]. This study included osseous metastatic non-rhabdomyosarcoma sarcoma patients treated to a dose of 40 Gy in five fractions and reported a 1- and 2-year LC rate of 82.5% each. Our LC rates, specifically for sarcoma histologies, were >90% at 1 and 2 years. There is a wide variability in the definition of LC and progression across several studies, given the persistent imaging abnormalities after SABR, especially bony metastases. We used the RECIST criteria for defining local control, and only progression within the radiation field was considered local progression.

The optimal BED_10_ for treating various pediatric tumors with SABR is not well defined. Prior studies have reported improved outcomes with a higher BED_10_ [16,22]. We also observed a significantly improved local control for patients who received a BED_10_ > 48 Gy. In addition, none of the patients who received a BED_10_ > 100 Gy had a local failure. We defined the cutoff at the median BED_10_ of 48 Gy to ensure an adequate number of events in both groups, given the small sample size. Only 8.9% of our patients received a BED_10_ ≥ 100 Gy. Of note, while 48 Gy was our median prescription dose to the PTV (equivalent to 30 Gy in five fractions), over 70% of our patients were treated with an SIB approach, with the GTV receiving a 25–50% higher BED_10_. Prior studies in metastatic sarcoma patients treated with spinal radiosurgery have shown a significant correlation of BED_10_ > 48 Gy with improved local control [23]. Singh et al. reported a 5% increase in 2-year LC for every 10 Gy increase in BED_10_ for sarcoma patients [20].

We also observed that patients with oligometastatic disease had a significantly longer PFS. This is consistent with several studies in the adult [15,24] as well as the pediatric population [21,25], suggesting improved PFS and OS in patients treated with total consolidation of metastatic disease with SABR. Elledge et al. reported an improvement in PFS (median, 9.3 months vs. 3.7 months; *p* = 0.03) as well as OS (median not reached vs. 12.7 months; *p* = 0.02) when all known sites of metastatic disease were consolidated with SBRT compared with partial consolidation [21]. Despite these reports, few patients with limited metastatic disease are treated with consolidative radiation therapy worldwide [26]. Surgery for metastatic disease was also associated with improved PFS, which supports the use of local consolidation with surgery when feasible in patients with metastatic pediatric cancers.

Tinkle et al. reported outcomes in 55 patients with 107 non-CNS lesions treated with SABR and observed a 1-year LC of 74.8% and an OS of 61% [27]. They observed that the radiographic response rates of bone and soft tissue lesions were at 90.6% and 76.7%, respectively. In our study, we did not see a difference in LC when stratified by metastasis location. We also observed poorer LC in patients who received concurrent chemotherapy during SABR. This might be due to a selection bias, as patients with higher-risk disease usually receive more aggressive systemic therapy.

Several prior studies reporting toxicity outcomes with SABR in pediatric patients have found it to be safe with a risk of acute and late toxicities [27,28,29,30]. The recent LITE-SABR meta-analysis reported an estimate pooled acute and late grade 3 to 5 toxicity rate of 2.9% [20]. We found similar results in our patients, with a grade 3 toxicity rate of 4.4% and no grade 4 or higher toxicities. The most common grade 3 toxicities seen were chronic pneumonitis and chest wall myositis, both of which were self-limited.

The use of SABR is increasing in prospective Children’s Oncology Group trials, including in ARST 1431 and AEWS1221, with doses between 20 and 40 Gy, depending on tumor histology and response to chemotherapy. Until these prospective trials report on these outcomes, single-institution studies will be valuable to ensure that SABR remains safe and effective with long-term follow-up.

This study is limited by its retrospective design and the inherent selection bias of patients who received SABR. One of the major limitations of this study is the lack of a matched non-SABR group as the comparison cohort for outcomes. Further prospective studies comparing the outcomes with SABR vs. conventional RT techniques in a randomized manner are warranted. We used historical controls to compare our outcomes. The definition of local failure and treatment response varies across studies, and this can impact the comparison of outcomes. In addition, the follow-up is variable, which limits the accurate evaluation of the toxicities. Another limitation is the heterogenous patient population inclusive of several radiosensitive and relatively radioresistant histologies. We were inclusive in our patient population to maintain the requisite number of events for meaningful results.

## 5. Conclusions

SABR is well tolerated in pediatric and AYA patients with local failure rates of <10% and a 1-year survival of 70%. LC was significantly higher for patients with sarcoma histologies and for those who received a BED_10_ > 48 Gy. The primary tumor histology did not impact the OS or PFS. Patients with oligometastatic disease and those who underwent surgery for the resection of a metastatic site had a significantly longer PFS. Further prospective studies to evaluate the role of SABR in metastatic pediatric cancer patients compared to systemic therapy alone are warranted.

## Figures and Tables

**Table 1:** Patient characteristics and treatment details.

Characteristic	N (%)
No. of patients	48
No. of courses	135
Courses per patient: median (IQR)	2 (1–17)
Age at diagnosis: median (IQR)	12.8 y (10–18 y)
Age at first SABR: median (IQR)	15.6 y (14–23 y)
Sex	
Male	34 (71)
Female	14 (30)
Lansky performance score	
90–100	25 (52)
<90	23 (48)
Primary tumor histology	
Sarcomas	30 (62)
Ewing’s sarcoma	12 (25)
Rhabdomyosarcoma	8 (17)
Osteosarcoma	6 (13)
Other sarcomas	4 (8)
Melanoma	2 (4)
Renal tumors	6 (13)
Wilms’ tumor	2 (4)
Renal cell carcinoma	2 (4)
Neuroblastoma	2 (4)
CNS tumors	8 (17)
Gliomas	6 (13)
Others	2 (4)
Treatment site (per course)	
Extraspinal osseous metastasis	44 (33)
Spine	39 (29)
Lung	32 (24)
Brain	12 (9)
Other soft tissue metastasis	8 (6)
Prior radiation (any site)	28 (64)
Reirradiation	24 (18)
Oligometastatic disease	77 (57)
RT dose: median (IQR)	30 Gy (25–35)
BED: median (IQR)	48 Gy (38–60 Gy)
Surgery	
Primary site	110 (81)
Metastatic site (at recurrence)	28 (2)
Concurrent chemotherapy	41 (30)

Abbreviations: IQR = interquartile range; SABR = stereotactic ablative radiation therapy; BED = biological equivalent dose.

**Table 2:** Univariate and multivariable analysis of factors affecting local control.

			Univariate Analysis			Multivariable Analysis		
Characteristic	N	Event	HR ^1^	95% CI ^2^	*p*-Value	HR ^1^	95% CI ^2^	*p*-Value
Age at RT	135	13	0.88	0.81, 0.97	0.008 *	0.88	0.80, 0.97	0.010 *
Sex					0.7			0.752
Male	108	11	—	—		—	—	
Female	27	2	0.74	0.16, 3.32		0.78	0.16, 3.75	
Sarcoma					0.010 *			0.213
No	32	7	—	—		—	—	
Yes	103	6	0.24	0.08, 0.72		0.47	0.14, 1.54	
Treatment Site					0.7	Not included		
Other osseous	44	4	—	—				
Spine	39	5	1.46	0.40, 5.37				
Lung	32	2	0.66	0.12, 3.59				
Brain	12	2	1.67	0.33, 8.58				
Lansky PS	135	13	0.98	0.95, 1.02	0.4	Not included		
Oligometastases					0.4	Not included		
No	58	4	—	—				
Yes	77	9	1.72	0.53, 5.61				
Number of lesions at SABR	135	13	0.81	0.58, 1.13	0.2	Not included		
BED (3rd vs. 1st quartile)	135	13	0.37	0.20, 0.68	0.001 *	0.44	0.29, 0.64	<0.001 *
BED					0.11	Not included		
≤48	98	12	—	—				
>48	37	1	0.20	0.03, 1.45				
Previous RT					0.4	Not included		
No	28	4	—	—				
Yes	107	9	0.64	0.20, 2.06				
Chemotherapy during SABR					0.048 *			0.008 *
No	94	6	—	—		—	—	
Yes	41	7	2.93	1.01, 8.48		5.56	1.56, 19.8	
Prior systemic therapy					0.3	Not included		
No	30	4	—	—				
Yes	105	9	0.53	0.17, 1.69				
Surgery for metastatic site					0.8	Not included		
No	107	10	—	—				
Yes	28	3	1.19	0.32, 4.48				

Abbreviations: RT = radiation therapy; PS = performance score; SABR = stereotactic ablative radiation therapy; BED = biological equivalent dose. ^1^ HR: hazard risk of local progression; ^2^ CI: confidence interval. * = *p*-value < 0.05.

## Data Availability

The research data are stored in an institutional repository and cannot be shared due to privacy issues and stipulations within our Institutional Review Board.

## Supplementary Materials

The following supporting information can be downloaded at https://www.mdpi.com/article/10.3390/cancers16112090/s1, Table S1: Univariate and multivariable analysis of factors affecting overall survival (OS). Table S2: Univariate and multivariable analysis of factors affecting progression-free survival (PFS). Table S3: Radiation-related toxicities. Figure S1: Risk of local progression stratified by sarcoma histology. Figure S2: Risk of local progression stratified by SABR site.
