# Supplementary material for: Disease Control and Toxicity Outcomes after Stereotactic Ablative Radiation Therapy for Recurrent and/or Metastatic Cancers in Young-Adult and Pediatric Patients
Source: Cancers (Basel). 2024 May 30;16(11):2090. doi: 10.3390/cancers16112090 (PMC11171376; doi:10.3390/cancers16112090)
Supplement: Supplementary file 1 [file cancers-16-02090-s001.zip › cancers-2990197-supplementary.pdf]

SUPPLEMENTARY

CONTENTS

**Page**	**Description**
1	Supplementary Table S1 (UVA and MVA of OS)
2	Supplementary Table S2 (UVA and MVA of PFS)
3	Supplementary Table S3 (Radiation-related toxicities)
4	Supplementary Figure S1 (local progression stratified by Sarcoma histology)
5	Supplementary Figure S2 (local progression stratified by SABR site)

Supplementary Table S1: Univariate and Multivariable analysis of factors affecting Overall Survival (OS)

			Univariate Analysis			Multivariable Analysis		
**Characteristic**	**N**	**Event N**	**HR[1]**	**95% CI[1]**	**p-value**	**HR[1]**	**95% CI[1]**	**p-value**
Age at RT	48	28	0.94	0.91, 0.97	**<0.001**	0.93	0.90, 0.97	**<0.001**
Sex					**0.043**			0.40
Female	34	19	—	—		—	—	
Male	14	9	1.75	1.02, 3.02		1.30	0.71, 2.41	
Sarcoma					0.67			
No	18	11	—	—		Not included		
Yes	30	17	0.89	0.51, 1.53				
Treatment Site					0.72			
Other osseous	13	8	—	—				
Spine	12	8	1.12			Not included		
Lung	8	5	0.64					
Brain	10	5	0.31					
Lansky PS	48	28	0.93	0.91, 0.95	**<0.001**	0.94	0.92, 0.97	**<0.001**
Oligometastases					**<0.001**			**<0.001**
No	17	14	—	—		—	—	
Yes	31	14	0.27	0.17, 0.44		0.25	0.14, 0.45	
BED > 48 Gy					**0.028**			0.23
≤48 Gy	36	24	—	—		—	—	
>48 Gy	12	4	0.52	0.29, 0.93		1.50	0.77, 2.89	
Previous RT					**0.040**			0.38
No	15	10	—	—		—	—	
Yes	33	18	0.58	0.35, 0.98		1.32	0.71, 2.47	
Chemotherapy during SBRT					**0.017**			0.11
No	37	20	—	—		—	—	
Yes	11	8	1.80	1.11, 2.91		1.52	0.91, 2.53	
Prior systemic therapy					0.72	Not included		
No	16	7	—	—				
Yes	32	21	1.12	0.61, 2.04				
Surgery for met					0.80			
No	31	19	—	—		Not included		
Yes	17	9	1.07	0.62, 1.86				

Supplementary Table S2: Univariate and Multivariable analysis of factors affecting Progression Free Survival (PFS)

			Univariate Analysis			Multivariable Analysis		
**Characteristic**	**N**	**Event**	**HR[1]**	**95% CI[1]**	**p-value**	**HR[1]**	**95% CI[1]**	**p-value**
Age at RT	135	101	1.02	0.99, 1.04	0.2	1.01	0.98, 1.04	0.41
Sex					0.2			0.07
Female	108	80	—	—		—	—	
Male	27	21	1.40	0.86, 2.27		1.62	0.96, 2.74	
Sarcoma					0.2	Not included		
No	32	24	—	—				
Yes	103	77	1.38	0.87, 2.19				
Treatment Site					**0.002**			0.07
Other osseous	44	32	—	—		—	—	
Spine	39	29	1.12	0.68, 1.86		0.85	0.7, 1.01	
Lung	32	25	0.64	0.37, 1.09				
Brain	12	8	0.31	0.14, 0.71				
Lansky PS	135	101	0.99	0.97, 1.01	0.2	Not included		
Oligometastases					**0.012**			0.32
No	58	36	—	—		—	—	
Yes	77	65	0.56	0.36, 0.88		0.78	0.48, 1.27	
BED (3rd vs. 1st quartile)	135	101	0.92	0.79, 1.07	0.2	Not included		
BED					0.553	Not included		
≤48 Gy	98	72	—	—				
>48 Gy	37	29	0.88	0.57, 1.36				
Previous RT					**0.031**			0.22
No	28	21	—	—		—	—	
Yes	107	80	0.56	0.34, 0.93		0.70	0.40, 1.23	
Chemotherapy during SBRT					0.3	Not included		
No	94	67	—	—				
Yes	41	34	1.24	0.82, 1.88				
Prior systemic therapy					0.5	Not included		
No	30	20	—	—				
Yes	105	81	1.19	0.73, 1.96				
Surgery for met					**0.027**			0.20
No	107	82	—	—		—	—	
Yes	28	19	0.58	0.34, 0.96		0.69	0.39, 1.21	

Supplementary Table S3: Radiation-related toxicities

Toxicity	Grade 1	Grade 2	Grade 3
**ACUTE**			
**Dermatitis**	5 (3.7%)	0	0
**Alopecia**	0	1 (0.7%)	0
**Acute pneumonitis**	2 (1.5%)	0	0
**LATE**			
**Chronic pneumonitis**	4 (3.0%)	2 (1.5%)	2 (1.5%)
**Neuropathy**	2 (1.5%)	0	1 (0.7%)
**Myositis**	0	0	2 (1.5%)
**Brachial plexopathy**	0	0	1 (0.7%)
**Radiation necrosis**	3 (2.2%)	2 (1.5%)	0
**TOTAL**	**16 (11.8%)**	**5 (3.7%)**	**6 (4.4%)**

Supplementary Figure S1: Risk of local progression stratified by Sarcoma histology

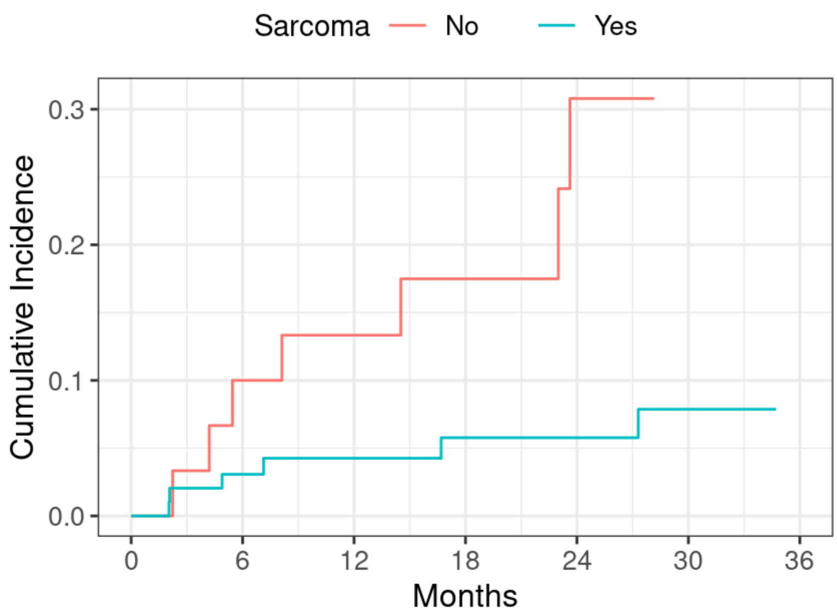

p=0.01

No

At Risk	32	24	19	6	3	1	1
Censored	0	2	3	9	10	11	11
Events	0	3	4	5	7	7	7

Yes

At Risk	103	66	41	28	22	16	10
Censored	0	5	18	25	30	32	37
Events	0	3	4	5	5	6	6

Supplementary Figure S2: Risk of local progression stratified by SABR site

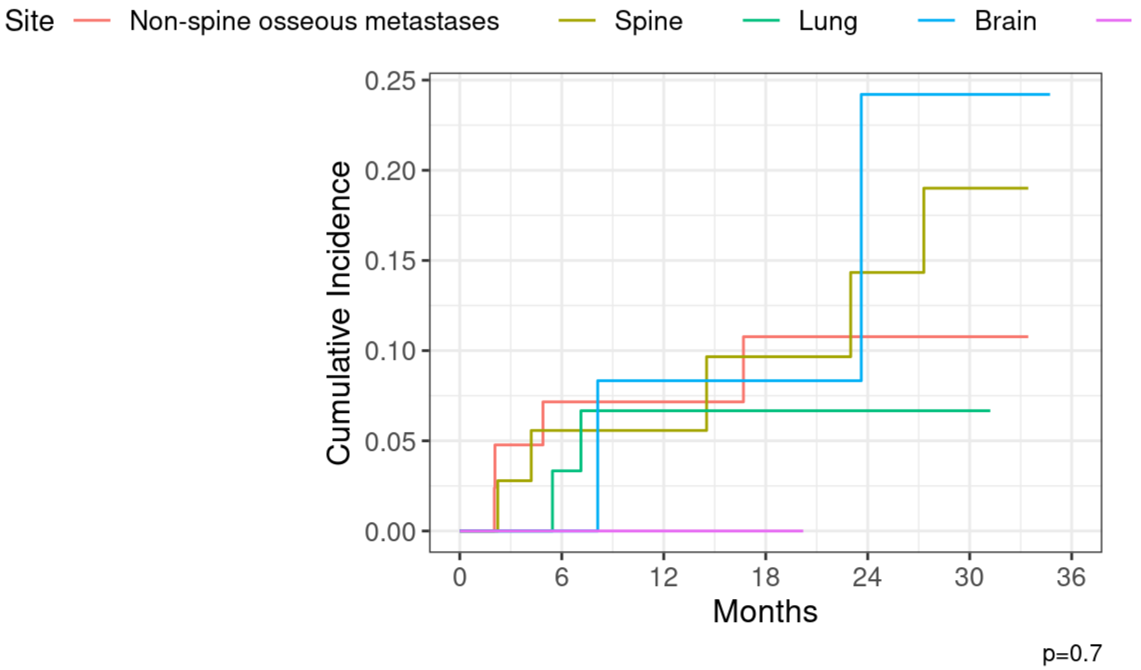

At Risk

	0	6	12	18	24	30	36
Non-spine osseous metastases	44	29	18	12	8	6	4
Spine	39	24	16	7	6	5	4
Lung	32	21	14	8	7	3	1
Brain	12	11	8	5	3	2	1
Other soft tissue metastases	7	4	3	1	0	0	0

Censored

	0	6	12	18	24	30	36
Non-spine osseous metastases	0	2	7	11	14	15	17
Spine	0	3	9	11	11	11	12
Lung	0	2	3	7	8	9	11
Brain	0	0	1	3	4	5	5
Other soft tissue metastases	0	0	1	2	3	3	3

Events

	0	6	12	18	24	30	36
Non-spine osseous metastases	0	3	3	4	4	4	4
Spine	0	2	2	3	4	5	5
Lung	0	1	2	2	2	2	2
Brain	0	0	1	1	2	2	2
Other soft tissue metastases	0	0	0	0	0	0	0
